# The role of long-term label-retaining cells in the regeneration of adult mouse kidney after ischemia/reperfusion injury

**DOI:** 10.1186/s13287-016-0324-1

**Published:** 2016-04-30

**Authors:** Xiangchun Liu, Haiying Liu, Lina Sun, Zhixin Chen, Huibin Nie, Aili Sun, Gang Liu, Guangju Guan

**Affiliations:** Department of Nephrology, The Second Hospital of Shandong University,Shandong University, Jinan, PR. China

**Keywords:** Label-retaining cells, Renal stem cells, Renal progenitor cells, Kidney regeneration, Ischemia/reperfusion injury, AKI

## Abstract

**Background:**

Label-retaining cells (LRCs) have been recognized as rare stem and progenitor-like cells, but their complex biological features in renal repair at the cellular level have never been reported. This study was conducted to evaluate whether LRCs in kidney are indeed renal stem/progenitor cells and to delineate their potential role in kidney regeneration.

**Methods:**

We utilized a long-term pulse chase of 5-bromo-2'-deoxyuridine (BrdU)-labeled cells in C57BL/6J mice to identify renal LRCs. We tracked the precise morphological characteristics and locations of BrdU^+^LRCs by both immunohistochemistry and immunofluorescence. To examine whether these BrdU^+^LRCs contribute to the repair of acute kidney injury, we analyzed biological characteristics of BrdU^+^LRCs in mice after ischemia/reperfusion (I/R) injury.

**Results:**

The findings revealed that the nuclei of BrdU^+^ LRCs exhibited different morphological characteristics in normal adult kidneys, including nuclei in pairs or scattered, fragmented or intact, strongly or weakly positive. Only 24.3 ± 1.5 % of BrdU^+^ LRCs co-expressed with Ki67 and 9.1 ± 1.4 % of BrdU^+^ LRCs were positive for TUNEL following renal I/R injury. Interestingly, we found that newly regenerated cells formed a niche-like structure and LRCs in pairs tended to locate in this structure, but the number of those LRCs was very low. We found a few scattered LRCs co-expressed Lotus tetragonolobus agglutinin (LTA) in the early phase of injury, suggesting differentiation of those LRCs in mouse kidney.

**Conclusions:**

Our findings suggest that LRCs are not a simple type of slow-cycling cells in adult kidneys, indicating a limited role of these cells in the regeneration of I/R injured kidney. Thus, LRCs cannot reliably be considered stem/progenitor cells in the regeneration of adult mouse kidney. When researchers use this technique to study the cellular basis of renal repair, these complex features of renal LRCs and the purity of real stem cells among renal LRCs should be considered.

## Background

Ischemia/reperfusion (I/R) is one of the leading causes of acute kidney injury (AKI) [1]. Approximately two million people die of AKI worldwide every year, despite the widely available renal replacement therapies [2, 3]. Interestingly, after acute injury, the kidney undergoes a quick regeneration to recover from renal functional failure by producing new cells. Thus, to develop targeted treatments for AKI, a clear understanding of the cell type contribution to the repair of injured kidney is a prerequisite. Whether the cells responsible for kidney regeneration are terminally differentiated tubular cells, pluripotent progenitor cells, or adult renal stem cells is still controversial [4–8].

Adult stem cells contribute to tissue repair in many organs such as skin, brain, heart, and gastrointestinal mucosa [9–11]. Adult nephron progenitors have been identified in zebrafish, but the role of adult stem/progenitor-like cells in mammalian renal repair remains unclear [12, 13]. The evidence supporting the role of adult stem/progenitor cells in the restoration of renal tubules after ischemic injury is derived from the presence of renal label-retaining cells (LRCs) in adult rodents. In 2003, Maeshima et al. first demonstrated the existence of renal progenitor-like tubular cells and their role in tubular regeneration by a 2-week chase using the label-retaining cell (LRC) technique [14]. Since then, the LRC technique has been improved by a long chase period to search for stem/progenitor cells in the adult mouse kidney. Using this technique, studies have been reported to demonstrate the role of renal stem/progenitor cells in the regeneration of ischemic renal injury at specific locations, such as renal papilla [15–17], S3 segment of proximal tubules [18], and the junction of cortex and outer medulla [19]. However, based on a lineage-tracing technique, recent studies showed that the surviving native tubular epithelial cells repaired the injured kidney without involving specialized progenitors [7, 20]. Thus, further studies are needed to resolve this controversy and to judge the real existence of renal stem/progenitor cells in the regeneration of adult kidney.

LRCs were recognized as rare stem and progenitor-like cells based on their slow-cycling, quiescent nature [21]. However, label retention by itself does not distinguish potential quiescent stem/progenitor cells from other less proliferative cell types that are labeled during the pulse period. Using the LRC technique in kidney, different studies demonstrated varying localization and function of LRCs during the repair of AKI [14–16, 18, 19, 22]. Rangarajan et al. revealed inconsistent localization of LRCs in adult kidney following deoxyuridine treatment at different dosages [23]. However, the complex biological features of LRCs in renal repair have never been elucidated. Considering the limitations of the LRC technique [24], we sought to evaluate whether these LRCs in kidney were indeed renal stem/progenitor cells and to delineate their potential role in kidney regeneration.

## Methods

### BrdU labeling

Male C57BL/6J mice were purchased from Vital River Laboratories (China). All animal procedures were given approval by the animal ethics committee of Shandong University (Jinan, China) and followed the Guide for the Care and Use of Laboratory Animals published by the US National Institutes of Health (NIH Publication No. 85–23, revised 1996). To investigate the long-term distribution of LRCs in mouse kidneys, postnatal mice were injected intraperitoneally with 5-bromo-2'-deoxyuridine (BrdU) (Sigma-Aldrich) at a dose of 50 μg/g twice daily, starting from 12 hours after birth to 3 days [25, 26]. The labeled pups were allowed to grow without a medical operation until eight-weeks old.

### Renal ischemia/reperfusion injury model

Eight-week-old BrdU-labeled C57BL/6J mice were subjected to renal bilateral I/R injury. Each group contained at least four mice. Briefly, mice were anesthetized with chloral hydrate (125 mg/kg, intraperitoneally) [27], and kidneys were exposed through a midline incision. The renal artery and vein were isolated from surrounding tissues and kidneys were subjected to ischemia by clamping bilateral renal pedicles with non-traumatic mirovascular clamps (Roboz Surg Instruments, USA) for 22 min [28, 29]. In the sham group, the renal pedicles were isolated without using clamps. After removing the clamps, reperfusion was verified and the incision was sutured and 1 ml of 37 °C saline was injected into the abdomen to supplement fluid loss. Mouse body temperature was maintained at 37 °C using a warming pad. Mice were sacrificed and blood and tissue samples were collected at 1, 2, 3, 4, 5, 6, 9, 14, and 28 days after I/R injury.

### Assessment of renal function

To assess renal function, serum creatinine (Scr) and blood urea nitrogen (BUN) levels were monitored. Serum was isolated from blood samples by centrifugation at 3000 rpm for 5 minutes. Scr and BUN were measured at the clinical laboratory of the Second Hospital of Shandong University using an automatic biochemistry analyzer (Beckman Instruments Inc., USA).

### Histology and immunohistochemistry

Renal tissues were fixed in 4 % paraformaldehyde, dehydrated and embedded in paraffin, and 5-μm-thick sections were cut using a microtome. For general histology, sections were stained with hematoxylin and eosin (H&E). BrdU immunohistochemistry was performed to determine the staining of LRCs [14, 25]. Primary antibodies against BrdU (Merck Millipore, USA, 1:50) were incubated with the sections overnight at 4 °C. Sections were washed with PBS, incubated with biotinylated goat anti-mouse IgG for 30 minutes, washed again and covered with horseradish peroxidase (HRP)-conjugated streptavidin (Zymed laboratories, USA) for 30 minutes. Next, a diaminobenzidine (DAB) solution was used to obtain a visible brown staining. Staining of Ki67-positive cells was performed in a similar manner. For the negative control, the primary antibody was omitted to estimate the specificity. Morphological images and examination of sections were captured by a light microscope (Nikon Instrument Inc.).

### Immunofluorescence

Renal tissues were embedded in OTC freezing medium (Sakura Finetek, Torrance, CA, USA) and sliced into 5-μm sections. The tissues were washed and blocked with 10 % goat serum for 30 minutes at room temperature, incubated with both the mouse monoclonal anti-BrdU (1:100) antibody and the rabbit polyclonal to Ki-67 antibody (Abcam, Cambridge, UK;1:100) or Lotus tetragonolobus agglutinin (LTA) antibody (Vector Labs, Berlingame, CA, USA;1:200) overnight at 4 °C. After washing with PBS, fluorescent secondary antibodies (rhodamine-conjugated goat anti-mouse IgG and FITC-conjugated goat anti rabbit IgG) were added to the sections in the dark for 1 hour at room temperature, and 4’,6-diamidino-2-phenylindole (DAPI) was used to stain nuclei. To observe cellular death in kidney, terminal deoxynucleotidyl transferase dUTP nick end labeling (TUNEL) (FragEL™ DNA Fragmentation Detection Kit-Fluorescent, Merck, Germany) was used by fluorescent staining the free 3' -OH termini of DNA [30]. For negative controls, the primary antibody was replaced with normal goat serum, which did not show positive staining, thus confirming specificity. Pictures of each section were obtained at × 200 or × 400 magnification with a fluorescence microscope (ECLIPSE-TS100, Nikon, Japan).

### Quantification of BrdU or Ki 67 or TUNEL-positive cells

The quantification of BrdU-retaining cells was performed by counting the number of positive nuclei in five selected fields of sections in a blinded manner under a light microscope at × 200. The average number was calculated as the number of BrdU-retaining nuclei per field. The fields of sections were generated from 3 ~ 4 separated BrdU-labeled mice per group, and the quantitative analysis was expressed by the average number of BrdU-labeled cells in the five random fields [14, 25]. Quantification of Ki 67 or TUNEL-positive cells was performed in a similar manner at × 400 magnification.

### Statistical analysis

Data are presented as means ± SEM. Differences between groups were analyzed by one-way ANOVA using SPSS 16.0. *P* < 0.05 was considered statistically significant.

## Results

### Restoration of mouse renal function and structure after I/R injury

**Fig. 1:**
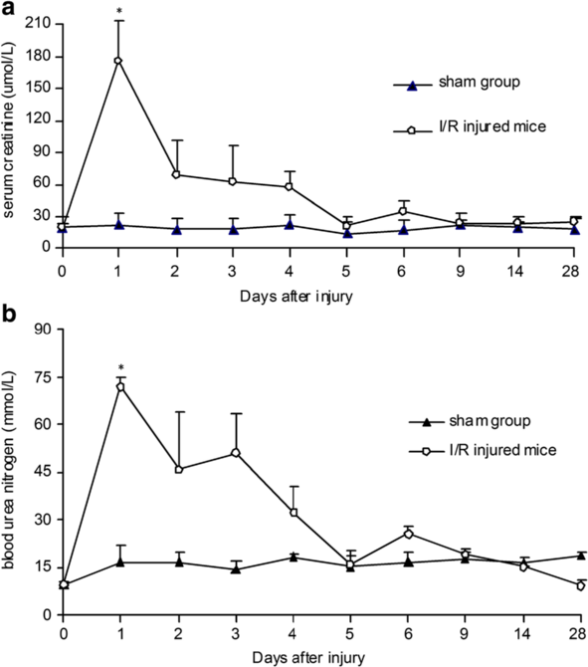
Restoration of renal function in C57BL/6J mice after I/R injury (*n* = 4 in each group). **a** Serum creatinine (Scr) level significantly increased one day after injury and was restored to normal gradually over time. **b** The peak value of blood urea nitrogen (BUN) was found at the end of the first day after injury, followed by return to normal levels (*P <* 0.05*,* I/R injured mice vs sham group). *I/R* ischemia/reperfusion

AKI activates pathways of cell death and cell proliferation. To investigate the natural healing process of injured kidneys, we observed the renal functional and structural changes in a mouse model of ischemic AKI. Scr and BUN were measured as markers of renal function at 0 (baseline), 1, 2, 3, 4, 5, 6, 9, 14, and 28 days after ischemia. We found significantly increased Scr levels on day 1. The peak value of Scr was 9-fold greater than the mean baseline value (Scr in μmol/L, 1 day after injury vs baseline, 175.93 ± 36.61 vs 19.53 ± 5.03, *P < 0.05*); it returned to normal within 9 days (Fig. 1a). The trend of BUN after I/R injury was consistent with Scr (Fig. 1b).

**Fig. 2:**
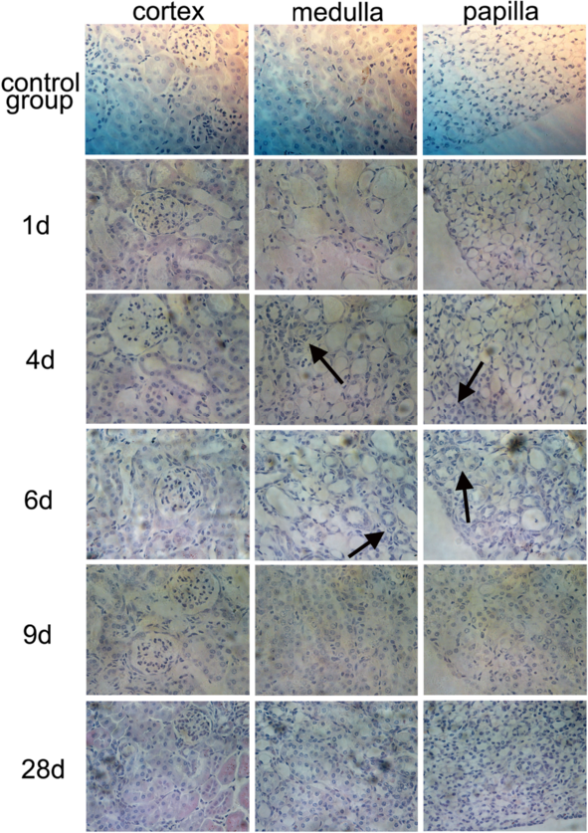
Renal histology in I/R injury. Kidney tissues from C57BL/6J mice with 22 min of bilateral renal ischemia or sham operation were collected on 1, 2, 3, 4, 5, 6, 9, 14, and 28 days and stained with H&E for histological examination. During the first 3 days of injury, kidney tissues showed severe tubular dilatation, loss of brush border, and sloughed debris in tubular lumen space. On day 4, the newly generated tubular cells increased rapidly. Nine days after I/R injury, the structure of damaged tissues showed normal histological architecture in cortex, medulla, and papilla (*arrows*, on day 4 and 6, indicate the special niche-like structure of newly restored tubular cells; magnification × 400). *I/R* ischemia/reperfusion, *H&E* hematoxylin and eosin

**Fig. 3:**
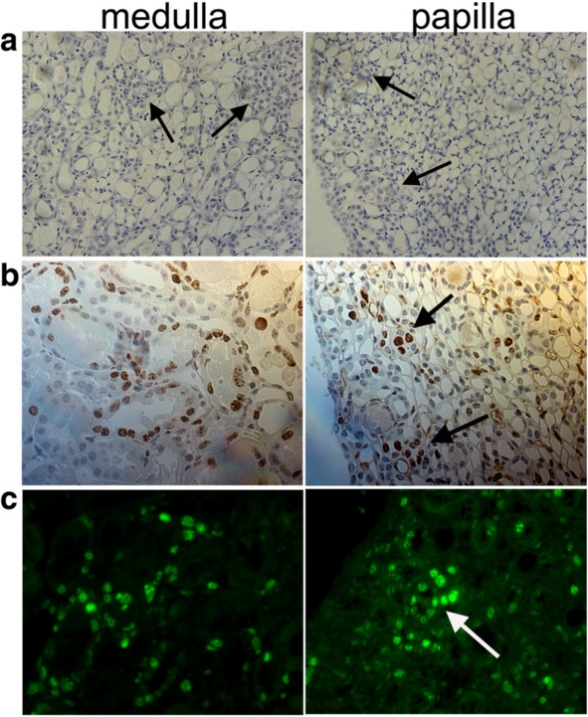
The newly regenerated cells formed a niche-like structure on day 4 during the repair process of I/R injury. **a** Kidney tissues stained with H&E for histological examination by magnification × 200; **b** Ki67-positive cells in medulla and papilla by immunohistochemistry (magnification × 400); **c** Ki67-positive cells in medulla and papilla by immunofluorescence (magnification × 400). *I/R* ischemia/reperfusion, *H&E* hematoxylin and eosin

To verify and localize the renal injury and regeneration directly, we examined the histological changes of kidney tissues in ischemic AKI by H&E staining. Twenty-four hours after I/R injury, we observed the typical renal tubular damage, such as severe tubular dilatation, loss of brush border, sloughed debris in tubular lumen space, and denuded basement membrane. The typical renal tubular damage lasted 3 days in renal cortex, medulla, and papilla (Fig. 2). On day 4, the newly generated tubular cells increased rapidly and formed a special niche-like structure of new cells (arrows in Fig. 2 and Fig. 3). The renal structure tended to restore rapidly within the next 3 ~ 5 days. Nine days after I/R injury, kidney tissues clearly showed normal histological architecture of renal cortex, medulla, and papilla (Fig. 2). These results indicated that the adult mouse kidney manifested natural regenerative capacity to repair I/R injured tubular cells.

### Distribution and morphological characteristics of long-term LRCs in normal mouse kidneys

**Fig. 4:**
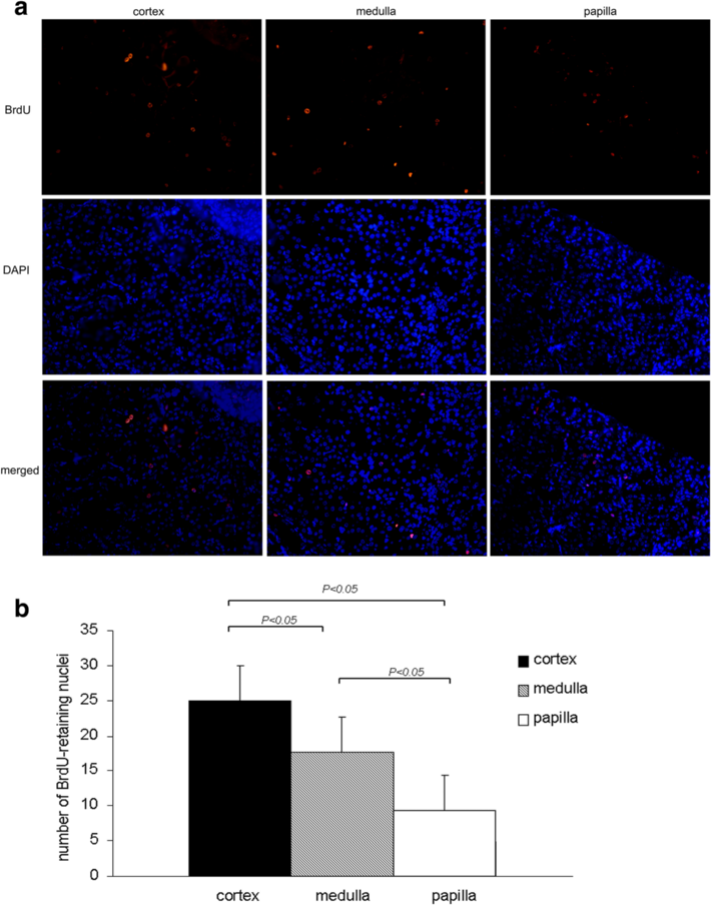
Localization of BrdU-retaining cells in 8-week-old mouse kidneys using immunofluorescence. **a** BrdU^+^ LRCs (red) scattered among normal adult kidneys; **b** BrdU-retaining cells in cortex, medulla, and papilla. BrdU-retaining cells were quantified by counting the number of positive nuclei in five randomly selected fields of sections under a fluorescence microscope (magnification × 200). *BrdU* 5-bromo-2'-deoxyuridine, *LRCs* label-retaining cells

**Fig. 5:**
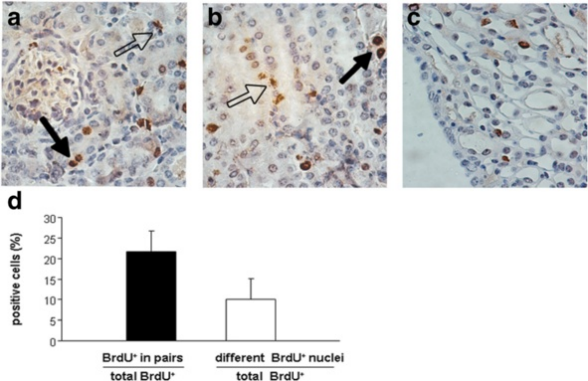
Localization of BrdU-retaining cells by staining with the DAB lectin in 8-week-old mouse kidneys. **a** In the cortex, most of the BrdU-retaining cells were located among the tubular cells, and a few of them were scattered in renal interstitium; **b** Localization of BrdU-retaining cells in the medulla; **c** BrdU-retaining cells on the renal papilla; **d** Quantitative analysis of BrdU+ LRCs located in pairs (*solid arrows*) and BrdU+ LRCs revealed nuclear fragmentation and disassembly (*dotted arrows*, magnification × 400). *BrdU* 5-bromo-2'-deoxyuridine, *DAB* diaminobenzidine, *LRCs* label-retaining cells

To investigate the nature and potential role of long-term LRCs further, we first identified the precise location of these cells in normal adult mouse kidneys. After an 8-week chase period with BrdU, we found that BrdU^+^ LRCs scattered among adult kidneys (Fig. 4 and Fig. 5). The difference in the number of these cells in cortex, medulla, and papilla was statistically significant (Fig. 3b*P <* 0.05). Most BrdU^+^ LRCs were located on the tubular cells, with a few scattered on renal interstitium (Fig. 5). These results indicated that the adult mouse kidney retained original cells that were quiescent in DNA synthesis within the first 3 days after birth.

In addition, to study the morphological characteristics of these cells directly, we observed BrdU^+^ LRCs under a microscope immunohistochemically. A few of the BrdU^+^ LRCs were located in pairs, and the number of these cells in total BrdU^+^ LRCs was 21.7 ± 1.5 % (Fig. 5 solid arrows, d). We observed that the nuclei of long-term BrdU^+^ LRCs exhibited different morphological characteristics in normal adult kidneys. Some of their nuclei were fragmented and disassembled, in approximately 10.1 ± 0.9 % of the BrdU^+^ LRCs (Fig. 5 dotted arrows, d). These fragmented and disassembled nuclei showed the variation of long-term LRCs in adult mouse kidneys, suggesting DNA damage or defect in these LRCs. In addition to slow-cycling cells, the damaged and non-proliferating cells may also be labeled by long-term BrdU^+^ LRCs in adult mouse kidney.

### Limited role of long-term LRCs in the regeneration of I/R injured kidney

**Fig. 6:**
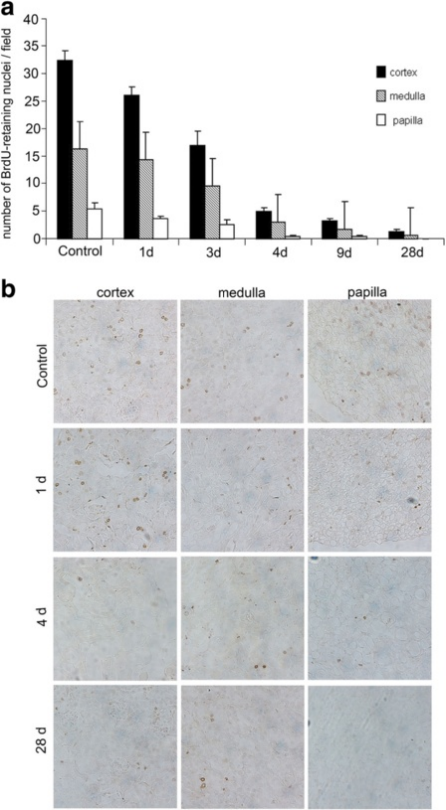
BrdU-retaining cells in mouse kidneys after I/R injury. After an 8-week chase period, the BrdU-labeled C57BL/6J mice were subjected to renal bilateral I/R injury. Kidney tissues were harvested 1, 2, 3, 4, 5, 6, 9, 14, and 28 days after I/R injury. BrdU-retaining cells were stained a visible brown color without using the hematoxylin. **a** Quantification of BrdU-retaining cells in I/R injured kidneys from the cortex, medulla, and papilla; LRCs diminished over time with renal functional recovery; four days after injury, the number of LRCs decreased dramatically. **b** Kidney sections from BrdU-stained cortex, medulla, and papilla showed the disappearance of LRCs after I/R injury. Upon restoration and recovery of the structure of the I/R injured kidney to normal within 1 month, LRCs were difficult to detect (magnification × 200). *BrdU* 5-bromo-2'-deoxyuridine, *I/R* ischemia/reperfusion, *LRCs* label-retaining cells

**Fig. 7:**
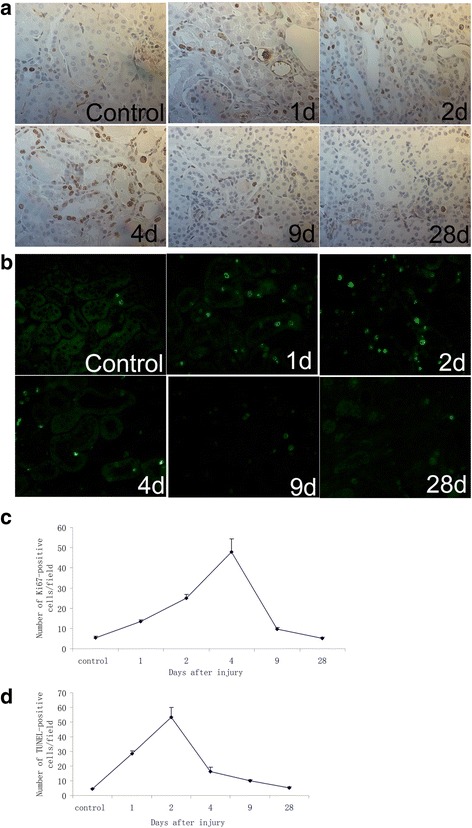
Analysis of the expression of Ki67-positive cells (**a**, **c**) and TUNEL-positive cells (**b**, **d**) in mouse kidneys after IRI (magnification × 400)

Eight weeks after BrdU-labeled mice underwent the renal I/R injury, we observed that the distribution of BrdU^+^ LRCs diminished over time with renal functional recovery. In the first 3 days after injury, the number of BrdU^+^ LRCs in medulla and papilla dramatically decreased while the majority of these cells in cortex decreased 4 days after injury (Fig. 6). For the repair process of acute renal I/R injury, both cell death and proliferation are essential components in the remodeling of tissue structures [31]. Thus, to investigate the contribution of LRCs in renal tubule regeneration after I/R injury, we first stained newly proliferating cells with Ki67, which specifically recognizes the proliferating cells [32], and stained apoptotic cells by TUNEL kit in our murine model. TUNEL^+^ cells peaked at 2 days after ischemic injury and a number of Ki67^+^cells were observed among tubular cells in ischemic kidneys 24 h after reperfusion (Fig. 7). At 4 days after the initial injury, Ki67^+^cells were significantly increased. Quantitative analysis of the number of TUNEL^+^ cells or Ki-67^+^ cells is shown in Fig. 7. These results indicated that apoptosis of tubular cells peaked at 2 days after ischemic injury, which is followed by a well-established repair phase of tubule regeneration via proliferation of tubular cells 3 ~ 5 days after injury. Therefore, we speculated that the quick disappearance of LRCs in the first 4 days after I/R injury may be due to both ablation of superfluous apoptotic cells and newly proliferating cells in the process of remodeling injured renal structures.

**Fig. 8:**
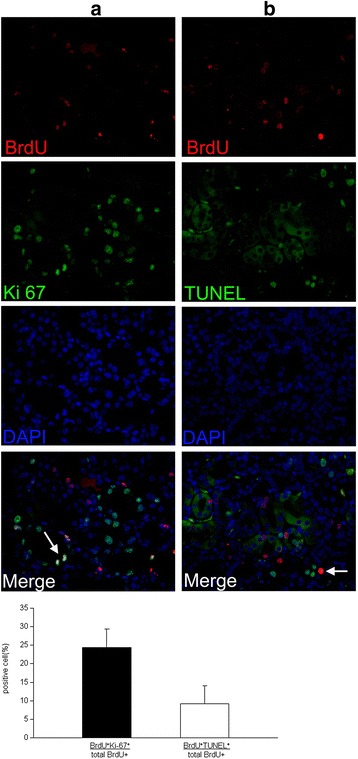
Contribution of BrdU^+^ LRCs in the regeneration of I/R injured mouse kidneys. **a** Double-immunofluorescence staining for BrdU (red) and Ki67 (green) to visualize the role of BrdU^+^ LRCs in cell proliferation 4 days after IRI. Newly generated cells formed a special niche-like structure (green), and only some of the BrdU^+^ LRCs co-expressed Ki-67 in this area (*arrows*). **b** Double immunofluorescence was used to determine BrdU^+^ LRCs undergoing cellular apoptosis on day 4. Scattered BrdU^+^ LRCs co-expressed TUNEL (*arrows*) (*arrows* indicate the double-stained location; magnification × 400). *BrdU* 5-bromo-2'-deoxyuridine, *LRCs* label-retaining cells, *I/R* ischemia/reperfusion

To verify this speculation, we further stained the co-localization of long-term LRCs and Ki-67^+^cells or TUNEL^+^cells in murine kidney after renal ischemia. On day 4, a period of renal recovery with more tubule proliferation and fewer cell deaths, we observed that not all BrdU^+^ LRCs were positive for Ki67 (Fig. 8a). BrdU^+^Ki67^+^ cells constituted only 24.3 ± 1.5 % of the total BrdU^+^ LRCs, and the majority of these BrdU^+^ LRCs occurred in pairs (arrows in Fig. 8a). BrdU^+^ LRCs located in pairs tended to distribute in the niche-like structure formed by newly proliferating cells. These results indicated that only a few of the total BrdU^+^ LRCs re-entered the cell cycle to contribute to the regeneration of I/R injured kidneys. In addition, we found that 9.1 ± 1.4 % of BrdU^+^LRCs co-expressed with TUNEL at day 4 after injury, and TUNEL^+^BrdU^+^cells were observed in isolated scattered BrdU^+^LRCs (arrows in Fig. 8b). This result demonstrated that long-term LRCs included cells with DNA fragmentation in the mouse kidney after injury and further indicated that not all the LRCs played a positive role in the regeneration process of I/R injured kidney.

### Phenotype of long-term LRCs in mouse kidney after I/R injury

**Fig. 9:**
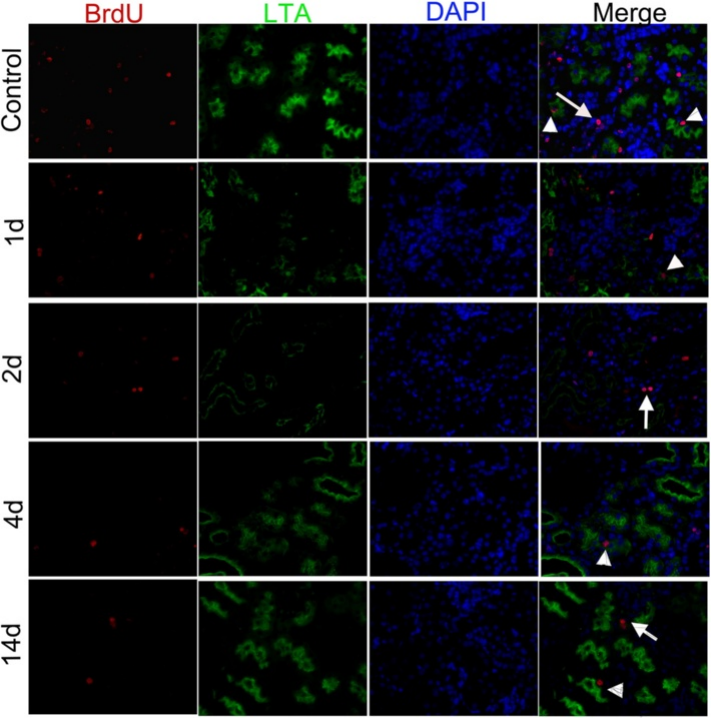
BrdU^+^ LRCs (red) co-expressed LTA (green) both in normal and I/R injured kidney (The *arrows* indicate LRCs in pairs, the *arrowheads* indicate isolated LRCs; magnification × 400). *BrdU* 5-bromo-2'-deoxyuridine, *LRCs* label-retaining cells, *LTA* Lotus tetragonolobus agglutinin, *I/R* ischemia/reperfusion

To further characterize the differentiation of LRCs, we examined the expression of Lotus tetragonolobus agglutinin (LTA), a marker of proximal tubular cell differentiation, in controls and I/R injured renal tissues. We found that some isolated scattered BrdU^+^ LRCs co-expressed LTA both in normal and I/R injured kidney, demonstrating that a few LRCs were in a differentiated state in the early phase of renal injury (arrowheads in Fig. 9). These results indicated that the scattered LRCs might not be the real stem-cell pool, and the regenerated immature cells might not be derived from long-term renal LRCs completely. Interestingly, BrdU^+^LRCs located in pairs did not co-express LTA in mouse kidney after I/R injury (arrows in Fig. 9). With the serious damage of I/R injured kidney on day 2, BrdU^+^LRCs seldom co-expressed LTA, indicating dedifferentiation of these LRCs at time points after I/R injury or migration of LRCs in kidney.

## Discussion

In this study, we demonstrated that the adult mouse kidney was capable of spontaneously restoring its functions from I/R injury, with only a small part of the total long-term BrdU^+^LRCs contributing to this regeneration. During the process, we found a few scattered LRCs exhibiting the characteristics of apoptotic cell death and cell differentiation in the early phase of tubular damage, which is inconsistent with the properties of stem/progenitor-like cells. Additionally, we first observed that the nuclei of long-term BrdU^+^ LRCs exhibited different morphological characteristics in normal adult kidneys, including fragmentation and disassembly of nuclei. Although we cannot rule out that LRCs in pairs are the real stem/progenitor cells in mouse kidney, we demonstrated that not all long-term LRCs represent the real niche of stem/progenitor cells in adult kidneys. Thus, our novel findings can serve as a reminder that putative renal stem/progenitor cell niches identified by the LRC technique in previous studies, such as renal papillae, need to be re-evaluated.

Although label-retaining cells have been observed in adult rodent kidney since 2003, the exact localization and the number of those cells are still controversial [23]. In the present study, we found that the number of long-term BrdU^+^ LRCs in cortex or medulla is much higher than that in papilla of 8-week old male C57BL/6J mice (Figs. 4 and 6). However, Rangarajan et al. showed a lower number of neonatal LRCs located in cortex or medulla and a higher number in the papilla [23]. One of the possible reasons for this discrepancy is mainly due to the sex differences in mouse between the studies. Rangarajan et al. used female C57BL/6J mice, while we used male mice for the present study. As we know, sex difference is found in responses to ischemia/reperfusion-induced acute kidney injury and females may attenuate the kidney injury [33–36]. In our study, the distribution of long-term LRCs also showed sex differences in the differing compartments of murine kidney compared with Rangarajan et al.’s study. Given that long-term LRCs were identified as somatic stem/progenitor-like cells, diverse distributions of those cells between different genders may be one potential explanation for the greater susceptibility to I/R renal injury in the male gender. Additionally, differences in methods of labeling administration, time interval of injection, species, and labeling markers may also result in inconsistent localization of LRCs between each of these studies [14, 17, 37]. For example, Oliver et al. labeled long-term LRCs by giving BrdU subcutaneously to 3-day-old Sprague-Dawley rats, while we labeled LRCs intraperitoneally, as in most studies on neonatal mice, twice daily from 12 hours after birth to 3 days. Renal tissue in neonatal rodent undergoes active development every day, especially within 7 days after birth [38, 39]. So the timing and methods of administration of deoxyuridine in rodent have an important effect on the localization of LRCs. In short, the above differences that exist in different studies should be considered when researchers estimate the localization of LRCs in adult kidney.

Among our results was the novel finding of the variation of long-term LRCs in adult mouse kidneys which was directly observed by a microscope immunohistochemically. Our results showed that the nuclei of long-term BrdU^+^LRCs were in pairs or scattered, fragmented or intact, strongly or weakly positive (Fig. 5), indicating different classes of long-term LRCs in adult mouse kidney. Thus, we observed biological characteristics of these LRCs in responses to I/R injury to clarify their roles in adult kidney. Although previous studies had observed that LRCs contributed to renal repair, the exact percentage of those LRCs counted by statistical analysis or the complex characteristics of long-term LRCs in the regeneration period of adult mouse kidney after I/R injury has never been reported [14, 15, 17, 19, 22]. In our study, we found that only 24.3 ± 1.5 % of total BrdU^+^LRCs co-expressed with Ki67 and 9.1 ± 1.4 % of BrdU^+^LRCs were positive for TUNEL at day 4 after injury (Fig. 8). Results showed newly regenerated cells did not completely derive from renal long-term LRCs in adult mouse kidneys. These results also showed only a small part of LRCs were responsible for repair, and most of the re-cycle LRCs were in pairs (Fig. 8a). Long-term LRCs exhibited complex characteristics, including cell proliferation, apoptosis and others that are unknown. Indeed, most of the BrdU^+^LRCs did not reenter the cell cycle after injury. Therefore, the proportion of long-term LRCs which can represent true stem/progenitor cells in the I/R injured kidney is also very low, consistent with the finding observed in the hematopoietic system [24]. Further studies will be needed to test the differentiation potency of these LRCs by isolated culture or biological markers to redefine these cells.

The majority of published studies about LRCs in renal repair did not report the severity of renal injury by monitoring renal function or structure in their animal models of ischemic AKI. Given that different severity of renal injury affects the spontaneous remodeling process in adult kidney [18, 29], we estimated both renal function and histological changes and analyzed both proliferating cells and apoptotic cells in our murine model. Results showed apoptosis of tubular cells peaked at 2 days after ischemic injury, which is followed by a repair phase of tubule proliferation that peeked on the 4th day (Fig. 7). During the process, the number of LRCs decreased with the repair process of I/R injured kidney, especially during the first 4 days after injury (Fig. 6). The causes of the loss of long-term LRCs after injury are still controversial. Oliver et al. attributed this phenomenon to reentry of LRCs into the cell cycle during the repair phase [15], whereas Humphreys et al. recently showed that cell division could not explain the loss of LRCs [7, 15]. We speculated that ablation of superfluous apoptotic cells may be one of the reasons for the quick disappearance of LRCs in the first 4 days after I/R injury.

Interestingly, we found that newly regenerated cells formed a niche-like structure (Fig. 3), and LRCs in pairs tended to locate in this structure (Fig. 8a). Most isolated scattered BrdU^+^ LRCs did not co-express Ki67 in the renal repair process. In this study, we also observed that a terminal differentiated proximal tubule marker, LAT, co-expressed in scattered BrdU^+^ LRCs in the early phase of renal injury (Fig. 9). This result further revealed the characteristic differentiation of a few long-term LRCs. Previous studies explained these differentiated LRCs as transit–amplifying (TA) cells, which are the early descendants of stem cells [14]. In our study, these differentiated LRCs were observed before the proliferating peak. Thus, these LRCs cannot be considered as TA cells.

The LRC technique is based on the assumption that somatic stem cells are quiescent or retain their original template DNA strands as they undergo slow cell cycle, compared with the majority of cells in the tissue. Although this technique has been used to identify stem cells in the mammary gland [40], neural stem cells [41] and the skin [42], it has its own limitations. On the one hand, stem cells in tissues cannot be completely detected by LRCs, challenging the recognition and quantification of stem cells using this method [43, 44]. On the other hand, not all LRCs are recognized as stem/progenitor cells. For example, Kiel et al. reported that fewer than 0.5 % of LRCs in the hematopoietic system are stem cells [24]. Another study showed that other long-lived, rarely cycling cells, such as leukocytes and endothelial cells, may be identified as LRCs [21]. Thus, label retention is neither sensitive nor specific for identifying stem/progenitor cells. Further, in our study, immunofluorescence showed that 9.1 ± 1.4 % of BrdU^+^ LRCs co-expressed cellular apoptosis marker in adult kidneys after the I/R injury (Fig. 8b). Immunohistochemical analysis revealed fragmentation and disassembly of 10.1 ± 0.9 % of the nuclei (Fig. 5). Therefore, we speculated that those LRCs may be arrested in the early phase of the cell cycle as a consequence of DNA damage or defect. The damaged and non-proliferating cells were also labeled, showing prolonged BrdU retention. Thus, our results further demonstrate that LRCs are not simple slow-cycling cells in adult kidneys, including cells with DNA damage or defect in the early phase.

Renal papilla is the structure composed of collecting ducts, loops of Henle, vasculature, and interstitial stromal cells [26]. Previous studies reported that LRCs of the renal papilla play a role in repair after ischemic injury and papillary LRCs were regarded as a stem cell niche in adult kidney [15–17, 45]. Recently, these findings have been questioned by using the genetic lineage analysis in kidneys [46]. Humphreys et al. have demonstrated that papillary LRCs do not contribute to the repair of I/R injured tubular cells. We also observed the papillary LRCs in our study, and our findings about complex biological characteristics of LRCs may provide a rational explanation for the inconsistent results.

The reason for the manifestation of different biological features by renal LRCs in the regeneration of I/R injured mice is also an unresolved issue. We speculate that since BrdU was injected over a 3.5-day period, characteristics of BrdU^+^LRCs might be varied during the active renal development in the first 7 days after birth. Renal LRCs may also include cells that are arrested in the early phase of the cell cycle as a consequence of DNA damage or defect. A recent study presented similar findings, suggesting that renal LRCs were not the same population with respect to different loading and chase periods [23].

In summary, we report novel findings that long-term renal LRCs exhibited multiple biological characteristics during the recovery from I/R injury in mouse kidneys. Renal LRCs do not totally represent the adult stem-cell population based on our observations of apoptotic LRCs and terminally differentiated LRCs in mouse kidneys. Although we cannot rule out the possibility that a small number of LRCs located in a special structure formed by newly proliferating cells in pairs are genuine stem progenitor cells, our findings demonstrate that long-term LRCs are not uniform in adult kidneys. This diversity in the LRC population should be considered in studies searching for real stem/progenitor cells in adult kidneys by the LRC technique. The purity of real stem cells among LRCs should be assessed in studies exploring the cellular basis of renal repair using this technique. Furthermore, our results also show that adult kidneys exhibit spontaneous recovery from acute renal injuries. Therefore, further studies are needed to clarify the types of renal cells mediating the repair and the remodeling of acutely injured kidneys to develop targeted interventions.

## Conclusions

Overall, this study revealed that long-term LRCs were not simple slow-cycling cells in adult kidneys, including cells with DNA damage or terminal differentiation in both normal and early I/R injury phase. Newly regenerated cells formed a niche-like structure to spontaneously repair injured renal cells, while only a small part of the LRCs in pairs tended to locate in this structure. Most isolated scattered LRCs did not reenter the cell cycle after injury, suggesting a limited role of long-term LRCs in the regeneration of I/R injured kidney. Thus, when researchers use the LRC technique to locate true stem/progenitor cells and to study the cellular basis of renal repair, these complex features of renal LRCs and the purity of real stem cells among renal LRCs should be considered.

## Abbreviations

AKI: acute kidney injury
BrdU: 5-bromo-2'-deoxyuridine
BUN: blood urea nitrogen
DAB: diaminobenzidine
I/R: ischemia/reperfusion
LRCs: Label-retaining cells
LTA: Lotus tetragonolobus agglutinin
Scr: serum creatinine
TUNEL: terminal deoxynucleotidyl transferase dUTP nick end labeling
